# Plasma Metabolomic Profiling after Feeding Dried Distiller’s Grains with Solubles in Different Cattle Breeds

**DOI:** 10.3390/ijms241310677

**Published:** 2023-06-26

**Authors:** Junjie Zhang, Tiantian Zhang, Duhan Xu, Mingming Zhu, Xiaofen Luo, Rong Zhang, Guangxia He, Ze Chen, Shihui Mei, Bijun Zhou, Kaigong Wang, Erpeng Zhu, Zhentao Cheng, Chao Chen

**Affiliations:** 1 College of Animal Science, Guizhou University, Guiyang 550025, Chinazhu13782701756@126.com (E.Z.); 2 Key Laboratory of Animal Diseases and Veterinary Public Health of Guizhou Province, College of Animal Science, Guizhou University, Guiyang 550025, China

**Keywords:** DDGS, GY, GC, plasma metabolome, metabolic pathways

## Abstract

Dried distiller’s grains with solubles (DDGS) are rich in nutrients and can enhance animals’ growth and immunity. However, there are few reports on the effects of a diet of DDGS on plasma metabolism and the related action pathways in domestic animals. In this study, groups of Guanling yellow cattle (GY) and Guanling crossbred cattle (GC) having a basal diet served as the control groups (GY-CG and GC-CG), and DDGS replacing 25% of the diet of GY and GC served as the replacement groups (GY-RG and GC-RG), with three cattle in each group. Plasma samples were prepared for metabolomic analysis. Based on multivariate statistical and univariate analyses, differential metabolites and metabolic pathways were explored. Twenty-nine significantly different metabolites (*p* < 0.05) were screened in GY-RG compared with those in GY-CG and were found to be enriched in the metabolic pathways, including choline metabolism in cancer, linolenic acid metabolism, and amino acid metabolism. Nine metabolites showed significant differences (*p* < 0.05) between GC-RG and GC-CG and were mainly distributed in the metabolic pathways of choline metabolism in cancer, glycerophospholipid metabolism, prostate cancer metabolism, and gonadotropin-releasing hormone (GnRH) secretion. These results suggest that a DDGS diet may promote healthy growth and development of experimental cattle by modulating these metabolic pathways. Our findings not only shed light on the nutritional effects of the DDGS diet and its underlying mechanisms related to metabolism but also provide scientific reference for the feed utilization of DDGS.

## 1. Introduction

Baijiu is widely deemed as the national liquor of China and one of the country’s leading industrial enterprises, with a large amount of liquor output every year; in 2022, the liquor output was 6,712,000 kiloliters (National Bureau of Statistics of China, 2023). A large number of byproducts are produced during the production of baijiu, including distiller’s grains (DGs), which, without proper disposal, can lead to the wasting of resources and to environmental pollution [1]. Large-scale development of animal husbandry in China has prompted a shortage of feed sources. Meanwhile, the high price of protein resources makes it necessary to seek alternative protein feed sources such as DGs [2]. DGs are subjected to a drying process to form DDGS; this process can improve their nutritional properties and extend their shelf life. DDGS are rich in many nutrients, including protein (25–33.3%), crude fiber (8.3–10.6%), carbohydrates (52.1–56.5%), amino acids (13.19–28.66%), minerals (29.5–33.5 mg/g), and various active factors [3], are suitable for development and utilization as ruminant feed resources, and could alleviate the lack of ruminant feed [4]. Some evidence has shown that supplementing a certain percentage of DGs into the diets of animals such as pigs (5–15%), sheep (30–50%), and cattle (20–30%) not only increases dry matter intake, average daily weight gain, and the feed conversion ratio, but also improves plasma biochemical indices [5,6]; however, there are few reports on the effect of feeding DGs on animal plasma metabolism. Therefore, understanding the underlying mechanisms of DGs and their nutritional effects on ruminant metabolism is critical for feed utilization of DGs.

Metabolomics is one of the latest omics, which can search for biomarkers through metabolic profiling to reveal biological reaction processes as well as the metabolic patterns in metabolic pathways, helping to provide important new ideas for animal nutrition research [7]. Liquid chromatography–mass spectrometry (LC-MS) is one of the most commonly used metabolomics techniques that allows for both rapid characterization of metabolites and accurate quantitative analysis [8]. To comprehensively examine the entire metabolome and analyze as many metabolic pathways as possible, untargeted screening is a good choice. Untargeted metabolomics is a common strategy for the discovery and identification of unknown suspect compounds [9]. Potential metabolites associated with metritis [10] and fatty liver disease [11] have been identified in dairy cows, and their underlying mechanisms have been explored by serum untargeted metabolomics analysis. Guanling yellow cattle (GY) are a fine local breed in Guizhou Province and geographically indicated for agricultural products with rough feeding resistance and strong adaptability, and its meat has a great tenderness [12]. Crossbred cattle of Simmental and yellow cattle have been used extensively to improve meat productivity and economic benefits in China [13]. To explore the metabolic pathways of the DDGS diet and its nutritional effects on different cattle breeds, the LC-MS method was adopted to analyze the plasma metabolites of GY and Guanling crossbred cattle (GC, Guanling yellow cattle crossed with Simmental cattle) after feeding on a DDGS diet. Then, certain metabolites and metabolic pathways associated with a DDGS diet were obtained; these may be helpful for understanding the nutritional value of DDGS in ruminants and laying foundations for the practical utilization of DDGS as feed resources.

## 2. Results

### 2.1. Quality Control of Untargeted Metabolic Profiling

Untargeted metabolomic analysis was performed on 12 test cattle by LC-MS. Base peak chromatograms of the quality control (QC) sample (a mixture of all the samples investigated) are illustrated in Figure S1, and all data were cross-validated by seven cycles to obtain principal component analysis (PCA) model plots for all plasma samples, in which QC samples were tightly clustered (Figure S2), implying that our analysis was repeatable and reliable. A total of 8314 substance peaks were detected by LC-MS in the samples, among which 1354 metabolites were identified, including 573 metabolites from the negative ion mode (ESI^−^) and 781 metabolites from the positive ion mode (ESI^+^), respectively (Figure S1c).

**Figure 1:**
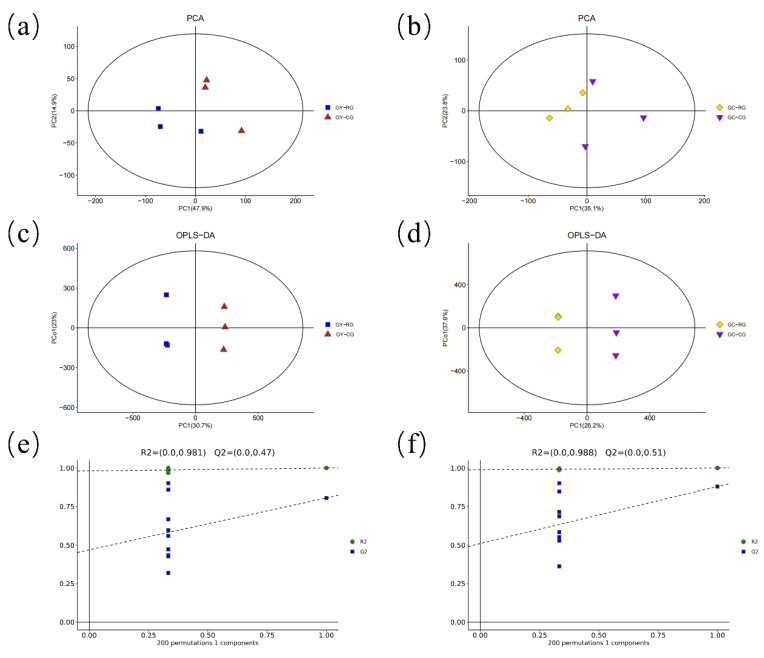
PCA and OPLS-DA score plots generated from plasma metabolic profiles. (**a**) Untargeted analysis in GY-RG and GY-CG. Cumulative fitness (R2 value) of the PCA model was 0.762. (**b**) Untargeted analysis in GC-RG and GC-CG. Cumulative fitness (R2 value) of the PCA model was 0.758. (**c**) OPLS-DA score plot of GY-RG and GY-CG, (R2X (cum) = 0.628, R2Y (cum) = 1, Q2 (cum) = 0.805). (**d**) OPLS-DA score plot of GC-RG and GC-CG, (R2X (cum) = 0.704, R2Y (cum) = 1, Q2 (cum) = 0.88). The PC1 and PC2 values in the figures represent the scores of each sample for principal components one and two, respectively. Each dot, square, or diamond on the plot represents a sample in the corresponding group. (**e**) OPLS-DA permutation test (200 times) in GY-RG and GY-CG. The results of the permutation test strongly indicate that the original model was valid (R2 intercept = 0.981, Q2 intercept = 0.47) (**f**) OPLS-DA permutation test (200 times) in GC-RG and GC-CG. The results of the permutation test strongly indicate that the original model was valid (R2 intercept = 0.988, Q2 intercept = 0.51). GY, Guanling yellow cattle; GC, Guanling crossbred cattle; CG, control group (the basal diet group); RG, DDGS 25% concentrate group.

Multivariate analysis was initially performed using the PCA scoring graphic and the processed data obtained from plasma metabolomics analysis in order to identify any systematic errors in the samples and observe the trends of each group. There was a clear trend of separation between the groups (Figure 1a,b). Orthogonal partial least squares discriminant analysis (OPLS-DA) was further performed to evaluate differences between the groups, showing that the corresponding two groups were clearly separated from each other, and R2 and Q2 values were higher than 0.46 (Figure 1c,d). Seven-fold cross-validation and permutation tests (*n* = 200) showed that the OPLS-DA model was stable and had strong predictive power (Figure 1e,f).

### 2.2. Differential Metabolite Screening

**Figure 2:**
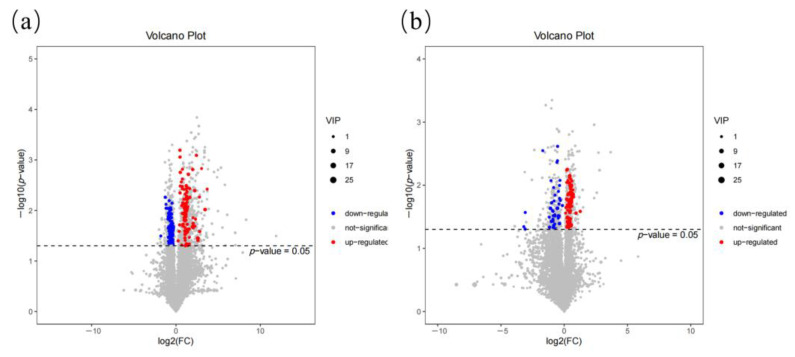
Volcano plots and hierarchical clustering of all detected features. The volcano plot was generated based on metabolites detected by the untargeted analysis in GY-RG vs. GY-CG (**a**) and GC-RG vs. GC-CG (**b**). A red dot represents an upregulated metabolite, a blue dot represents a downregulated metabolite, and a grey dot represents a nonregulated metabolite. The horizontal dotted line represents the level of significance (*p* < 0.05).

To visualize the results of the fold change analysis of 8314 substance peaks for quick identification of significant differential metabolites, a volcano plot was drawn by transferring the fold change (FC) value of each substance peak to log2 (FC), transferring the *p*-value (*p*  =  0.05) of Student’s *t*-test to –log10 (*p*-value) and simultaneously meeting the variable importance in projection (VIP) values > 1 for the first principal component of the OPLS-DA model and in the *p* < 0.05 for the *t*-test (Figure 2). The number of upregulated metabolites was greater than the number of downregulated metabolites for the pairwise comparisons (Guanling yellow cattle DDGS replacement group vs. Guanling yellow cattle control group and Guanling crossbred cattle DDGS replacement group vs. Guanling crossbred cattle control group, GY-RG vs. GY-CG and GC-RG vs. GC-CG) (Figure 2), with 29 significantly differential metabolites (12 were upregulated, 17 were downregulated) as the important different metabolites, including lipids as well as lipid-like and organic compounds (Table 1). Additionally, nine metabolites (four upregulated, five downregulated) showed significant differences and were super-classified into lipids and lipid-like molecules (LysoPC(22:5(4Z,7Z,10Z,13Z,16Z)), capsidiol, PS(17:2(9Z,12Z)/20:0), SM(d18:1/16:1), PC(15:0/22:2(13Z,16Z)), testosterone, monocyclic botryococcane), and unclassified (LysoPC(P-18:1(9Z)), PC(16:0/O-1:0)) (Table 1).

### 2.3. Metabolic Pathways Analysis

**Figure 3:**
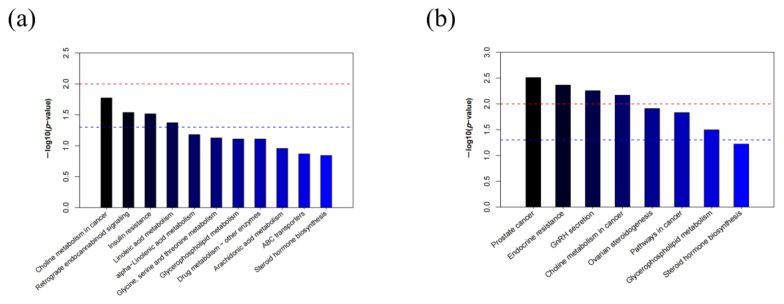
Metabolic pathway analysis of the differentially expressed metabolites in GY-RG vs. GY-CG (**a**) and GC-RG vs. GC-CG (**b**). In (**a**,**b**), the red dotted line indicates a *p*-value of 0.01, and the blue dotted line indicates a *p*-value of 0.05. The color of the bar graph from light to dark indicates that the *p*-value decreases in order, and when the top of the bar is higher than the blue line, a pathway is considered to be significant.

To further reveal the metabolic differences between GY-RG and GY-CG, GC-RG and GC-CG, a Kyoto Encyclopedia of Genes and Genomes (KEGG) pathway enrichment analysis was performed for the twenty-nine and nine significantly different metabolites, respectively. The results showed that the pathways significantly enriched (*p* < 0.05) in GY-RG compared with those in GY-CG were choline metabolism in cancer, retrograde endocannabinoid signaling, insulin resistance, and linoleic acid metabolism (Figure 3a). The pathways that were significantly enriched in GC-RG compared with those in GC-CG were prostate cancer metabolism, endocrine resistance, GnRH secretion, choline metabolism in cancer, ovarian steroidogenesis, pathways in cancer, and glycerophospholipid metabolism (Figure 3b). Among them, choline metabolism in cancer was the shared significantly enriched metabolic pathway.

## 3. Discussion

The application of DGs as feed ingredients in livestock production has been proven to effectively improve the growth performance and slaughter performance of cattle, sheep, and other ruminants and also to promote rumen fermentation and intestinal development [14]. Dehydration of DGs to obtain DDGS further improves their utilization rate and can alleviate the problems of resource waste and environmental pollution [14]. The present study showed that the DDGS diet, through the modulation of metabolic pathways including fatty acid metabolism, amino acid metabolism, glycerophospholipid metabolism, choline metabolism in cancer, prostate cancer metabolism, and GnRH secretion, could exert pro-growth and anti-inflammatory effects. A total of 29 metabolites with significant differences were screened in GY-RG compared with those in GY-CG, and these differential metabolites mainly involved fatty acyls, steroids, organic acids, and organic compounds. Pathway analysis based on the KEGG and Human Metabolome Database (HMDB) mainly included choline metabolism in cancer, fatty acid metabolism, and amino acid metabolism. Nine metabolites with significant differences were screened in GC-RG compared with those in GC-CG, mainly involving glycerophosphates, steroids, and sphingolipids. Pathway analysis based on KEGG and HMDB databases included choline metabolism in cancer, glycerophospholipid metabolism, prostate cancer and GnRH secretion. Among them, choline metabolism in cancer was the shared significantly enriched metabolic pathway. Differences in growth performance and microbial composition among breeds of cattle have been reported to affect metabolic pathways [15], and the difference in plasma metabolism between the two groups of test cattle after feeding with DDGS in this experiment may be attributable to these differences.

The PC(20:0/18:3(6Z,9Z,12Z)) and LysoPC(22:5(4Z,7Z,10Z,13Z,16Z)), which are important metabolites of choline metabolism in cancer, were significantly downregulated in GY-RG compared with GY-CG and GC-RG compared with GC-CG, respectively (Figure 2). It has been suggested that abnormal choline metabolism is associated with tumor formation and cancer development, and various intermediate enzymes, metabolites, and transporter proteins involved in choline metabolism are currently considered biomarkers in the progression of various cancers [16]. As one of the most important final products of choline metabolism, PC(20:0/18:3(6Z,9Z,12Z)), also known as phosphatidylcholine (PtdCho), is the most abundant phospholipid in mammalian cell membranes and is a major component of eukaryotic cell membranes [17]. PtdCho is synthesized from free choline via the Kennedy pathway and catabolized to GPC (glycerophosphocholine) and 1-acyl-GPC, which is then converted to free choline, thus completing the choline cycle. The PtdCho has diagnostic and prognostic value in cancer [18]. The LysoPC(22:5(4Z,7Z,10Z,13Z,16Z)), also known as lysophosphatidylcholine (LPC), is a well-known inflammatory lipid that is upregulated in a variety of inflammation-related diseases and is the most abundant lysophospholipid in plasma [19]. LPC can be decomposed into GPC and fatty acids (FAs) catalyzed by acyl-protein thioesterase 1 and then decomposed into choline and glycerophosphoric acid by GPC, thus regulating choline metabolism in cancer and glycerophospholipid metabolism [20]. Glycerophospholipids are the major lipid components of cell membranes; they play important roles in cell proliferation, differentiation, and apoptosis [21]. Fluctuations in glycerophospholipid content reflect disturbances in lipid metabolism and are an important biological indicator [22]. In recent years, several studies have shown that lipids are increased in a variety of diseases in cattle, including mastitis, metritis, and retained placenta [23,24]. This experiment suggests that a DDGS diet may regulate choline metabolism in cancer and glycerophospholipid metabolism by downregulating PC(20:0/18:3(6Z,9Z,12Z)) and LysoPC(22:5(4Z,7Z,10Z,13Z,16Z)), which is beneficial to the growth and development of organisms and has some inhibitory effects on the development of cancers and inflammation.

In this work, the fatty acid metabolic pathway of GY-RG compared with that of GY-CG was significantly enriched, including linoleic acid metabolism and α-linolenic acid metabolism. The concentration of PtdCho, a starting substrate for linoleic acid metabolism and α-linolenic acid metabolism, showed a significant decrease in the experimental group, which may affect linoleic acid and α-linolenic acid metabolism (Figure 2). Essential fatty acids (EFAs) have been shown to have beneficial effects on various diseases in animals (humans and most mammals, including ruminants) that cannot synthesize EFAs (such as alpha-linolenic acid (ALA) and linoleic acid (LA)) on their own and must obtain them through food [25]. The immunomodulatory effects of EFAs are mainly achieved through the synthesis of eicosanoids (e.g., prostaglandins, leukotrienes, and thromboxanes) by EFAs. The major n-6 FAs are LA and arachidonic acid; the major n-3 FAs are ALA and its metabolites eicosapentaenoic acid, docosapentaenoic acid, and docosahexaenoic acid [26]. Changes in the ratio of n-6 and n-3 FAs directly affect the production of mediators associated with the secretion of pro-inflammatory cytokines, such as tumor necrosis factor-α, IL-1β, and IL-6 [27]. LA is the most abundant polyunsaturated fatty acid (PUFA), and as an n-6 PUFA, it is an essential nutrient for many organisms [28]. LA is essential for normal growth and development, cell function and signaling, and immune responses [29]; it can also be metabolized to form inflammatory mediators associated with liver injury, and excessive intake of it can cause metabolic liver disease due to hepatic lipid peroxidation [30]. However, the direct role of LA in metabolic health remains controversial and needs to be further investigated [31]. ALA is an n-3 PUFA that may reduce the risk of various diseases, such as cardiovascular disease, cancer, and inflammatory diseases, to some extent [32]. It also has some adverse effects, such as involvement in processes such as oxidative stress and inflammation [33]. The concentration of PtdCho, a starting substrate for linoleic acid metabolism and α-linolenic acid metabolism, was significantly decreased, indicating that DGs could promote the absorption of PtdCho by the organism, thereby reducing oxidative stress and strengthening immunity.

Glycine betaine was significantly downregulated in GY-RG compared with that in GY-CG. The serine, glycine, and threonine metabolic pathways were enriched. Glycine betaine is one of the most widely distributed betaines in nature; it can be synthesized by choline dehydrogenase and betaine aldehyde dehydrogenase catalyzed by the body and can also be obtained from the diet, in foods such as spinach, wheat bran, shrimp, and amaranth seeds [34]. Several studies have reported that glycine betaine plays an important role in osmolarity regulation, alongside nutritional and health issues [35,36]; it also has a protective role in a variety of pathologies, including several liver diseases, cancer, hemophilia, and aging [37]. In choline metabolism, choline is first oxidized by choline oxidase to betaine aldehyde, which is further oxidized to betaine; then, betaine is sequentially demethylated to form dimethylglycine, sarcosine, and glycine, which are associated with anti-inflammatory properties and immune regulation [38]. Then, the concentration of glycine regulates the concentration of serine and threonine, which play a key role in the synthesis of essential cellular metabolites [39]. Glycine betaine was significantly downregulated, probably because the DDGS diet promoted its absorption in the small intestine, resulting in a decrease in plasma levels of glycine betaine. It is suggested that the DDGS diet may have growth-promoting and anti-inflammatory effects.

Testosterone concentration showed a significant downward trend in GC-RG compared with that in GC-CG. Testosterone, an important substrate for the synthesis of androgen receptor (AR), can synthesize AR not only directly but also through dihydrotestosterone, which is catalyzed by 3-oxo-5-alpha-steroid 4-dehydrogenase 2. AR is the steroid hormone nuclear receptor that drives the majority of prostate cancer [40]; it plays a key role in the development and progression of prostate cancer, especially in its evolution to advanced stages. Thus, targeting AR signaling is a pillar of advanced prostate cancer treatment strategies [41]. Our results suggest that a DDGS diet may reduce AR synthesis by decreasing the concentration of testosterone, thus acting as a preventive agent against prostate cancer. Testosterone is also one of the two initiation products of the GnRH secretion pathway, which indirectly inhibits the activity of the kisspeptins (Kiss1) metastasis suppressor and affects the synthesis of Kiss1. Kiss1 can strongly depolarize GnRH neurons, thus affecting GnRH synthesis [42]. GnRH is synthesized in the hypothalamus of vertebrates and is a key endocrine regulator that drives the hypothalamus–pituitary–gonad axis and ultimately activates the reproductive endocrine network. In addition to being involved in the regulation of gonadotropins, sexual behavior, olfactory signaling, fetal growth and development, and the formation and function of the immune system [43], GnRH can also improve the spermatogenic ability and sperm quality of bulls, induce estrus and ovulation in cows, and enhance the possibility of conception [44,45]. Testosterone was significantly downregulated, suggesting that the DDGS diet may affect the metabolism of GnRH and subsequently the endocrine regulation of the body, thereby modulating growth and enhancing the reproductive performance and development of the fetus. Because the GnRH secretion pathway involves positive feedback regulation and complex metabolism, further experiments are needed to investigate the effect of DDGS on this pathway and its potential mechanisms.

## 4. Materials and Methods

### 4.1. Subjects Studied

Maotai-flavored distiller’s grains were obtained from Kweichow Moutai Group in Moutai, Renhuai, China, and a drum dryer was used for the drying treatment before feeding. Six GY and six GC, each in good health and of similar age (18 months old), weighing 250 ± 25 kg, were provided by the beef cattle fattening farm of the Guanling County Yellow Cattle Group. The criteria for good health were negative results on a brucellosis tiger red plate agglutination test and test tube agglutination test, negative results on bovine foot-and-mouth disease type O and bovine foot-and-mouth disease type A real-time fluorescence quantitative PCR tests, and no obvious trauma, mental depression, or loss of appetite. The test cattle were ear-marked one by one and randomly divided into the Guanling yellow cattle control group, the Guanling crossbred cattle control group, the Guanling yellow cattle DDGS replacement group, and the Guanling crossbred cattle DDGS replacement group (GY-CG, GC-CG, GY-RG, and GC-RG); there were three cattle in each group. The composition and nutrient content of the diets are shown in Supplementary Table S1. The diets were mixed to make a full mixed diet, and the cattle were fed twice daily (9:00 a.m. and 4:30 p.m.). The cattle were given free water and grouped into pens throughout the experimental period.

### 4.2. Sample Preparation

On the 60th day of positive test feeding, overnight fasting was performed, and jugular vein blood from each test cow was collected into anticoagulation tubes in the early morning. After fresh blood was stored at 4 °C for approximately 1 h, plasma samples were obtained by centrifugation at 4000× *g* for 10 min, and QC samples were prepared by mixing equal volumes of extracts from all samples. The blood samples were removed and thawed at room temperature, 100 μL of the samples was pipetted; 10 μL of internal standard (L-2-chlorophenylalanine, 0.3 mg/mL; methanol) was added, vortexed, and shaken for 10 s; and 300 μL of protein precipitant methanol–acetonitrile (V:V = 2:1) was added, vortexed and shaken for 1 min; the samples were extracted by ultrasonication in an ice-water bath for 10 min and left for 30 min at −20 °C. They were then centrifuged for 10 min (13,000 rpm, 4 °C), and 300 μL of supernatant was evaporated into an LC-MS injection vial. The samples were re-dissolved with 400 μL of methanol–water (V:V = 1:4) (vortex for 30 s, sonication for 3 min) and then left to stand for 2 h at −20 °C before centrifugation for 10 min (13,000 rpm, 4 °C) and aspiration of 150 μL of supernatant with a syringe, using a 0.22 μm organic phase pinhole filter. Finally, they were transferred to an LC injection vial and stored at −80 °C until LC-MS analysis was performed.

### 4.3. LC–MS Analysis

The LC-MS analysis of plasma samples was performed using a liquid–liquid mass spectrometry system consisting of an ACQUITYUPLCI-Class ultra-performance liquid-phase tandem VION IMS Q-Tof high-resolution mass spectrometer. A 1 μL aliquot of plasma samples was injected onto a column ACQUITY UPLC™ BEH C18 (50 mm × 2.1 mm i.d., 1.7 μm; Waters Corporation, Milford, MA, USA). Mobile phase A was 0.1% formic acid in water, and B was an acetonitrile/methanol solution (2/3) containing 0.1% formic acid (*v*/*v*); the flow rate was 0.4 mL/min. The conditions for UPLC separation and ESI-VION IMS Q-TOF detection are shown in Tables S2 and S3. The QC samples were used to evaluate the reproducibility and reliability of the LC-MS system, which contains metabolic information inside all samples and is used to evaluate the stability of the mass spectrometry system. The ion peaks with a relative standard deviation of >0.4 for the QC group samples were removed, and the stability of the system was evaluated by PCA. The PCA model plot was obtained after seven cycles of cross-validation to detect whether the QC samples were closely clustered together; the plot was then used to judge the stability of the instrument’s detection.

### 4.4. Data Processing and Analysis

Before data pre-processing for pattern recognition, the raw data were processed by metabolomics software Progenesis QI v2.3 (Nonlinear Dynamics, Newcastle, UK) for baseline filtering, peak identification, integration, retention time correction, peak alignment, and normalization with the following main parameters: precursor tolerance: 5 ppm/10 ppm (self-built library), product tolerance: 10 ppm/20 ppm (self-built library), production threshold: 5%. Compound identification based on accurate mass numbers, secondary fragmentation, and isotope distribution, using the HMDB, Lipidmaps (v2.3) and METLIN databases as well as self-built libraries, was used for characterization. For the extracted data, the ion peaks with missing values (0 value) >50% within the group were deleted, and the 0 value was replaced by half of the minimum value. The compounds obtained from the characterization were screened according to the score of the compound characterization results, with a screening criterion of 36 points out of 60; results below 36 points were considered inaccurate and were deleted from the characterization results. Finally, the positive and negative ion data were combined into a data matrix containing all the information extracted from the original data that can be used for analysis, and a multivariate statistical analysis was carried out using SIMCA 14.0 (Umetrics AB, Umeå, Sweden). An unsupervised PCA was first used to examine both the metabolic differences between groups and the individual metabolic differences between samples. To maximize the differences between groups within the model, a supervised OPLS-DA was performed on metabolites between groups. In addition, to prevent overfitting of the model, the quality of the model was examined by response permutation testing with 200 responses; then, univariate analyses were performed on the sample data, which consisted of a multiplicative analysis of variance and a *t*-test. The FC was obtained by fold analysis of variance to calculate the fold change in the expression of certain metabolites between the two groups; the *p*-value was obtained using a *t*-test, and then volcano plotting was performed using Origin2020 (OriginLab, Northampton, MA, USA) to visualize the *p*-value and FC in order to facilitate the rapid identification of metabolites with significant differences. A multivariate statistical analysis and univariate analysis were combined to screen the differential metabolites between groups.

### 4.5. Putative Identification of Important Metabolites

The VIP values were obtained according to the OPLS-DA model, and potential metabolites were found based on VIP > 1 and *p* < 0.05. To support metabolite identification, the following databases were used: the HMDB (http://www.hmdb.ca/, accessed on 10 June 2022), Lipidmaps (v2.3), METLIN (http://metlin-scripps.edu/, accessed on 10 June 2022), and the KEGG (http://www.genome.jp/KEGG/, accessed on 10 June 2022). The KEGG was used to annotate the main differential metabolites. The pathway enrichment analysis was performed using the KEGG ID of the differential metabolites, and the hypergeometric test was applied to find if a pathway was significantly enriched (*p* < 0.05 of enrichment analysis) in the differential metabolites compared to the whole background, with *p* < 0.05 of enrichment analysis as the threshold. Moreover, the pathway topology was analyzed based on the relative betweenness centrality with a bos taurus pathway library of KEGG database.

### 4.6. Statistical Analysis

All continuous variables were expressed as the mean ± standard deviation (M ± SD). The differences between the groups were analyzed using Student’s *t*-test, and *p* < 0.05 was statistically significant. All statistical analyses were performed using SPSS 26.0 (Chicago, IL, USA). The PCA and OPLS-DA were performed to reveal the global metabolic changes between the control and stroke groups, and SIMCA 14.0 was used. A validation plot was used to assess the validity of the OPLS-DA model using seven-fold cross-validation and permutation tests (*n* = 200).

## 5. Conclusions

Our results showed that the DDGS diet, through the modulation of metabolic pathways including fatty acid metabolism, amino acid metabolism, glycerophospholipid metabolism, choline metabolism in cancer, prostate cancer metabolism, and GnRH secretion, could exert pro-growth and anti-inflammatory effects. These findings contribute to further understanding of the nutritive and regulatory functions of the DDGS diet for different breeds of cattle and its underlying mechanisms of action, thus providing a theoretical basis for the large-scale application of DDGS as a feed resource.

## Figures and Tables

**Table 1:** The significantly different metabolites in positive (pos) and negative (neg) ion modes.

GY-RG Compared with GY-CG						
Nos.	Metabolites	VIP	*p*-Value	FC	Ion Mode	Super-Class
1	6-Methylmercaptopurine	17.849	0.026	2.338	neg	Organoheterocyclic compounds
2	Benzaldehyde, p-amino-, thiosemicarbazone	6.266	0.021	3.992	neg	Unclassified
3	C16 Sphinganine	6.238	0.023	0.720	pos	Lipids and lipid-like molecules
4	PC(20:0/18:3(6Z,9Z,12Z))	5.615	0.021	0.79	pos	Lipids and lipid-like molecules
5	6-Thioxanthine	4.608	0.035	6.028	neg	Unclassified
6	Sodium tetradecyl sulfate	3.125	0.035	0.674	neg	Organic acids and derivatives
7	Betaine	2.644	0.029	0.705	pos	Organic acids and derivatives
8	Nuatigenin 3-beta-D-glucopyranoside	2.555	0.012	0.594	neg	Lipids and lipid-like molecules
9	Ethyl 1-(propylthio)propyl disulfide	2.553	0.018	4.114	neg	Organosulfur compounds
10	Diethyl disulfide	2.164	0.0134	2.073	neg	Organosulfur compounds
11	Norfloxacin	1.906	0.021	0.551	pos	Organoheterocyclic compounds
12	L-Acetylcarnitine	1.780	0.012	0.565	pos	Lipids and lipid-like molecules
13	Scillirosidin	1.773	0.025	0.598	pos	Lipids and lipid-like molecules
14	PC(18:2(9Z,12Z)/20:1(11Z))	1.573	0.0424	0.754	pos	Lipids and lipid-like molecules
15	4-Ethynylaniline	1.484	0.049	2.151	pos	Unclassified
16	2E,4E,6Z-Nonatrienal	1.454	0.0221	4.081	neg	Lipids and lipid-like molecules
17	CP 339818	1.426	0.013	0.527	pos	Unclassified
18	5-(Methylthio)-2-[(methylthio)methyl]-2-pentenal	1.413	0.004	2.592	neg	Organic oxygen compounds
19	PC(18:1(11Z)/18:3(6Z,9Z,12Z))	1.402	0.037	0.659	neg	Lipids and lipid-like molecules
20	O-Cresol	1.400	0.042	0.645	pos	Benzenoids
21	N-oleoyl glutamic acid	1.316	0.011	0.667	pos	Lipids and lipid-like molecules
22	5,7-octadienoic acid	1.226	0.0148	4.995	pos	Lipids and lipid-like molecules
23	Palmatine	1.206	0.008	0.441	pos	Unclassified
24	11-Dehydrocorticosterone	1.191	0.032	0.287	pos	Lipids and lipid-like molecules
25	Armillaripin	1.182	0.029	0.590	pos	Lipids and lipid-like molecules
26	2,3-dinor Thromboxane B1	1.141	0.014	4.058	neg	Unclassified
27	8-Hydroxyguanine	1.127	0.012	0.630	pos	Organoheterocyclic compounds
28	2E,7-Octadienoic acid	1.046	0.020	4.785	pos	Lipids and lipid-like molecules
29	(7R)-7-(5-Carboxy-5-oxopentanoyl)aminocephalosporinate	1.033	0.008	2.377	pos	Organoheterocyclic compounds
GC-RG Compared with GC-CG						
Nos.	Metabolites	VIP	*p*-Value	FC	Ion Mode	Super-Class
1	LysoPC(P-18:1(9Z))	5.757	0.043	1.458	pos	Unclassified
2	LysoPC(22:5(4Z,7Z,10Z,13Z,16Z))	3.239	0.046	0.554	neg	Lipids and lipid-like molecules
3	Capsidiol	2.862	0.043	0.565	pos	Lipids and lipid-like molecules
4	PS(17:2(9Z,12Z)/20:0)	2.721	0.023	0.472	neg	Lipids and lipid-like molecules
5	SM(d18:1/16:1)	2.368	0.037	0.494	pos	Lipids and lipid-like molecules
6	PC(15:0/22:2(13Z,16Z))	2.095	0.013	1.417	pos	Lipids and lipid-like molecules
7	Testosterone	1.660	0.026	0.541	pos	Lipids and lipid-like molecules
8	Monocyclic botryococcane	1.133	0.041	1.228	pos	Lipids and lipid-like molecules
9	PC(16:0/O-1:0)	1.064	0.033	1.271	pos	Unclassified

Note: VIP, variable influence on projection; FC, fold change; GY, Guanling yellow cattle; GC, Guanling crossbred cattle; CG, control group (the basal diet group); RG, DDGS 25% concentrate group.

## Data Availability

The authors confirm that the data supporting the findings of this study are available within the article and the Supplementary Materials.

## Supplementary Materials

The following supporting information can be downloaded at: https://www.mdpi.com/article/10.3390/ijms241310677/s1.
