# Supplementary material for: Plasma Metabolomic Profiling after Feeding Dried Distiller’s Grains with Solubles in Different Cattle Breeds
Source: Int J Mol Sci. 2023 Jun 26;24(13):10677. doi: 10.3390/ijms241310677 (PMC10341706; doi:10.3390/ijms241310677)
Supplement: Supplementary file 1 [file ijms-24-10677-s001.zip › ijms-2309149-supplementary/Supplementary files/Supplementary figures.pdf]

Supplementary figures

Figure S1. Base peak chromatograms (BPCs) of the quality control (QC) sample and global metabolites identified.

Figure S2. PCA score plots generated from serum metabolic profiles.

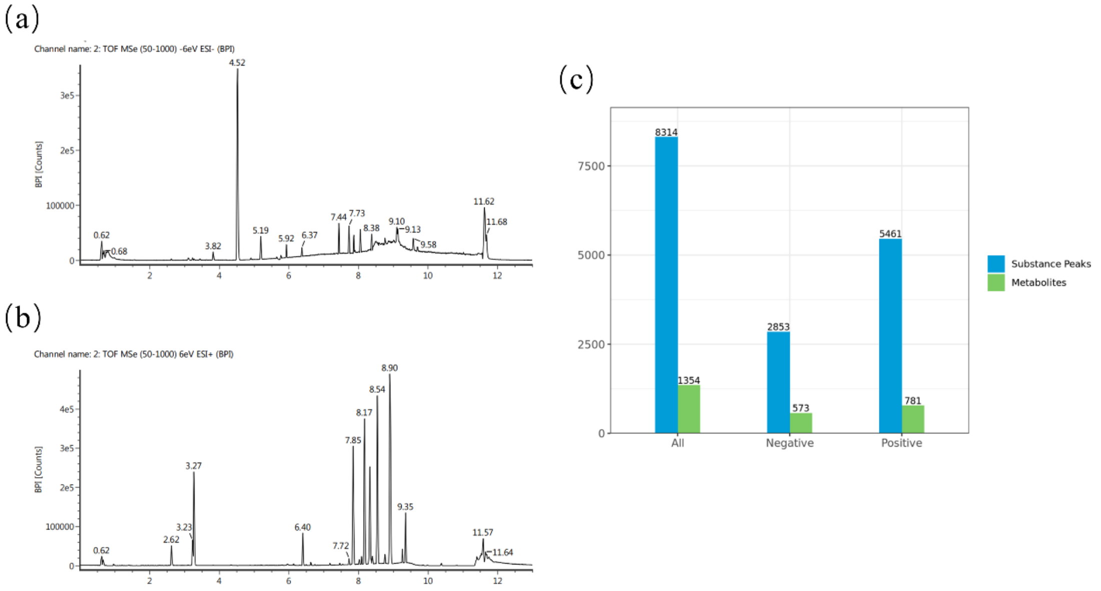

Figure S1. Base peak chromatograms (BPCs) of the quality control (QC) sample and global metabolites identified. (a) BPCs of negative electrospray ionization (ESI^+), (b) BPCs of positive ESI (ESI^-). (c) The number of substance peaks determined and metabolites identified.

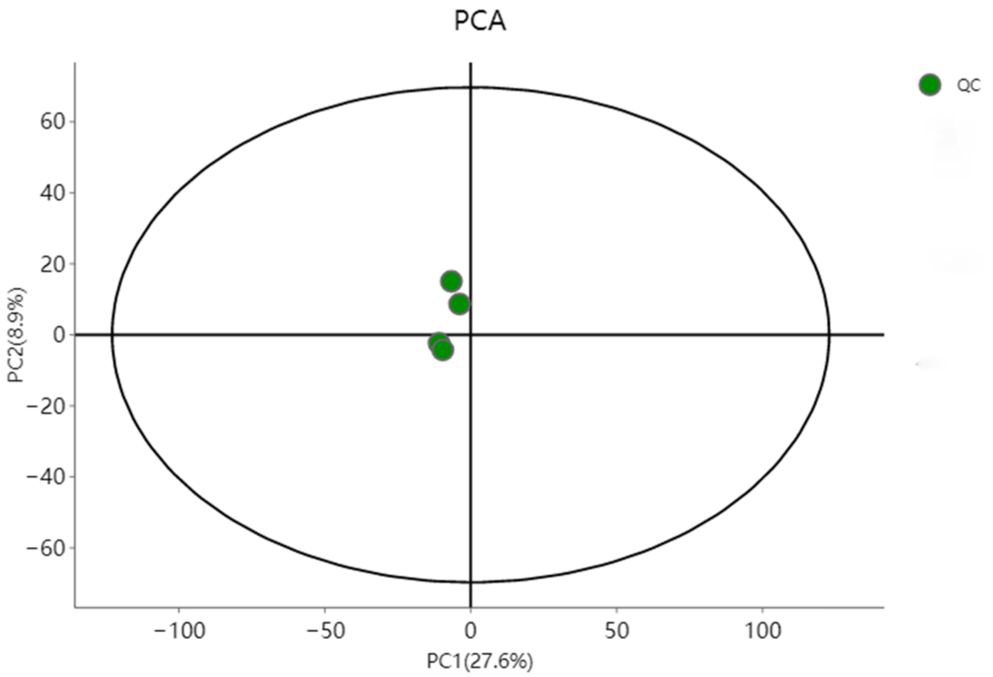

Figure S2. PCA score plots generated from serum metabolic profiles. Score scatter plot of QC samples with PC1 and PC2.
